# Combined Artificial Intelligence Approaches Analyzing 1000 Conservative Patients with Back Pain—A Methodological Pathway to Predicting Treatment Efficacy and Diagnostic Groups

**DOI:** 10.3390/diagnostics11111934

**Published:** 2021-10-20

**Authors:** André Wirries, Florian Geiger, Ahmed Hammad, Andreas Redder, Ludwig Oberkircher, Steffen Ruchholtz, Ingmar Bluemcke, Samir Jabari

**Affiliations:** 1 Spine Center, Hessing Foundation, Hessingstrasse 17, 86199 Augsburg, Germany; florian.geiger@hessing-stiftung.de (F.G.); ahmed.hammad@hessing-stiftung.de (A.H.); andreas.redder@hessing-stiftung.de (A.R.); 2 Center for Orthopaedics and Trauma Surgery, Philipps University of Marburg, Baldingerstrasse, 35043 Marburg, Germany; oberkirc@med.uni-marburg.de (L.O.); ruchholt@med.uni-marburg.de (S.R.); 3 Neuropathological Institute, University Hospitals Erlangen, Schwabachanlage 6, 91054 Erlangen, Germany; Ingmar.Bluemcke@uk-erlangen.de (I.B.); samir.jabari@uk-erlangen.de (S.J.)

**Keywords:** artificial intelligence, supervised, unsupervised, machine learning, methodology, back pain, spine, conservative

## Abstract

Patients with back pain are common and present a challenge in everyday medical practice due to the multitude of possible causes and the individual effects of treatments. Predicting causes and therapy efficien cy with the help of artificial intelligence could improve and simplify the treatment. In an exemplary collective of 1000 conservatively treated back pain patients, it was investigated whether the prediction of therapy efficiency and the underlying diagnosis is possible by combining different artificial intelligence approaches. For this purpose, supervised and unsupervised artificial intelligence methods were analyzed and a methodology for combining the predictions was developed. Supervised AI is suitable for predicting therapy efficiency at the borderline of minimal clinical difference. Non-supervised AI can show patterns in the dataset. We can show that the identification of the underlying diagnostic groups only becomes possible through a combination of different AI approaches and the baseline data. The presented methodology for the combined application of artificial intelligence algorithms shows a transferable path to establish correlations in heterogeneous data sets when individual AI approaches only provide weak results.

## 1. Introduction

Artificial Intelligence (AI) is gaining more and more influence in medical care. However clinical disease presentations are complex and prediction of the progress of a disease for the individual patient is often difficult. Patients with back pain are a typical example as the causes of pain are diverse and complex ranging from simple muscular spasms to malignant tumors or serious injuries. Thus the perception and processing of the complaints as well as the extent of therapeutic success through medical measures show a high interindividual variability [1].

Finding the right cause of back pain and better estimation of success rates of a conservative therapy would help to propose a suitable treatment. This could facilitate the initiation of individually appropriate therapy and the determination of a suitable diagnosis without the excessive use of costly and time-consuming, often invasive diagnostics. An insufficient or too excessive care of patients could be avoided.

In recent years, there have been increasingly promising efforts to improve medical care in the field of back pain and spinal therapy through the application of artificial intelligence [2,3,4,5,6]. Typically, two approaches are followed to establish an AI dealing with medical issues. Supervised AI performs learning on repetitive, comparable procedures, allowing the prediction of defined target variables. In non-supervised AI approaches, patterns are searched for in data sets to apply them to the course of a disease. In the case of spinal conditions, however, the predictions have so far been limited, irrespective of the AI approach used, as the full spectrum of available technical possibilities has so far only been exploited in a few cases.

To further improve AI prediction, we have developed a combined AI approach. For this purpose, a dataset of 1000 patients with spinal complaints who underwent standardized conservative therapy was utilized. The individual efficiency of this treatment was first examined in a supervised AI approach. Secondly a non-supervised clustering was performed. Then a combination of both approaches by supervised prediction of clusters was established. The joint application of all three approaches then enabled the prediction of diagnostic groups through the analysis of cluster subgroups. This entire process exemplifies how a combination of AI approaches can enable diagnostic prediction.

## 2. Materials and Methods

### 2.1. Patient Population

Prospectively collected data of 1000 consecutive patients presented at the Spine Center of the Hessing Foundation in Augsburg, Germany, between August 2018 and January 2020 were used. All patients suffered from back pain and received standardized inpatient conservative therapy. All patients were treated according to the same therapy sequence over 5 days, whereby the therapy modules provided were individually adaptable.

Complaints were assessed at baseline and at the end of therapy using the Oswestry Disability Index (ODI) and a visual analogue scale (VAS), separately for leg and back pain. The ODI is based on a questionnaire and ranges from 0 to 100. Higher scores in the ODI indicate a higher disability [7]. VAS is measured on a 100 mm visual analogue scale and is reported as a number between 0 and 10, with 10 indicating highest possible pain level. All of the patients provided informed written consent to the use of their data. The data collection took place within the framework of participation in the German Spine Registry. For use in the present project, patient data were anonymized at the time of inpatient discharge. The complete data set was processed in a translational approach together with the digital pathology and AI working group at the University Hospitals Erlangen, Germany, to establish the combined AI method presented here.

### 2.2. Course of Standardised Conservative Therapy

All patients received a combination of different therapeutic measures over 5 days including daily specific spinal infiltrations (epidural, periradicular or facet infiltrations). Additionally, daily physiotherapeutically guided exercises, balneo-physical applications and learning of pain-coping strategies were carried out in individual and group therapies.

### 2.3. Content and Structure of Database

Basic demographic data, as well as the ODI value along with separate VAS score for leg and back pain for every patient were assessed on the day of admission. The ODI was re-assessed on the day of discharge following inpatient treatment. The data collected from the 1000 patients were divided into three quality categories. As a result, 100% complete data sets were available for 427 patients. In 211 cases there was an insufficient response regarding the ODI questionnaires (more than one question has not been answered). The remaining 362 records had multiple incomplete data.

The mean age of the patients was 62.8 years and ranged from 18 to 95 years. 55.2% of patients were female, 44.8% male, 0% diverse. In total, 37.1% of patients had a BMI above 30 kg/m^2^ and were thus classified as obese. A total of 31.2% of patients stated that they were regular smokers. In total, 28.9% of the patients had previously undergone spinal surgery.

### 2.4. Supervised Prediction of Treatment Efficiency

The data collected from the 1000 patients were stored in a csv file format that was read by the pandas python package (pandas v. 0.23.1 [8]; python 3.6.7 [9]). Plotting of correlation matrix (matplotlib v. 2.1.2 [10] and seaborn v. 0.8.1 [11]), density distributions, histograms of various parameters and basic statistical operations were performed on the dataset.

Then, to predict the patient outcome after initial treatment during hospital stay, we defined the ODI Score after treatment to be our target predictive value, hence the machine learning problem was a linear regression problem. Applying recursive feature elimination, weighing of feature importance and analysis of intercorrelating features, half of the parameters within the csv file for a given patient were dropped to reduce complexity (feature selector v. 1.0.0). The final parameters used during the machine learning are shown in Table 1.

After identification of categorical variables, these were implemented in a neural net. The rest were continuous variables that have been collected in a separate array as a separate input to the model. The model used had multiple categorical inputs processed via an embedding layer and one input for the continuous variables. All inputs were concatenated and processed through two additional hidden layers with rectified linear activation functions and a subsequent linear output at the last layer. The Keras framework (v. 2.2.4) [12] with tensorflow backend (v.1.12.0) [13] was used to model the network architecture and perform network training.

### 2.5. Cluster Analysis and Prediction

Firstly, in a non-supervised approach, the whole dataset was investigated. Therefore we used a Python implementation of an algorithm for dimensionality reduction (Uniform Manifold Approximation and Projection for Dimension Reduction; UMAP) [14] to visualize our data. By using the Hierarchical Density-Based Spatial Clustering of Applications with Noise (HDBSCAN) [15] python implementation we identified three unique clusters within our dataset of 1000 patients and assigned the identified cluster labels to the dataset.

Secondly, we trained a simple shallow neural net to predict the cluster labels assigned. Eventually we identified the most important features within our dataset contributing to the class label hence the cluster they were assigned to by using the python implementation of SHapley Additive exPlanations (SHAP) [16] which is a game-theoretic approach to explain the output of any machine learning model. Finally, these features were overlayed and assigned to the plot showing the distinct clusters from our patient data.

### 2.6. Combination of AI Approaches

Machine learning is used in the supervised AI approach, on the one hand, to predict the ODI score at the end of conservative therapy and on the other hand to predict the cluster group of the non-supervised AI approach as described above.

**Figure 1:**
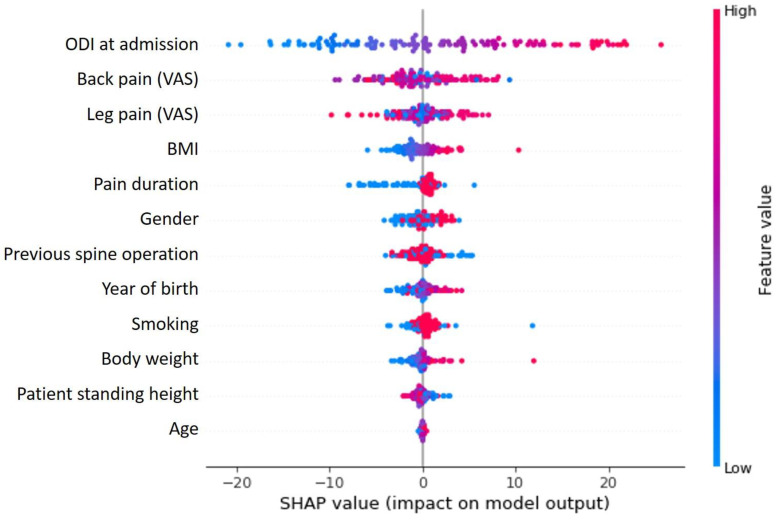
SHAP value analysis shows the relationship between variables and their influence on clustering results. The ODI at the time of admission clearly has the greatest influence, followed by the other parameters (VAS for back and limb pain) used to describe complaints.

**Figure 2:**
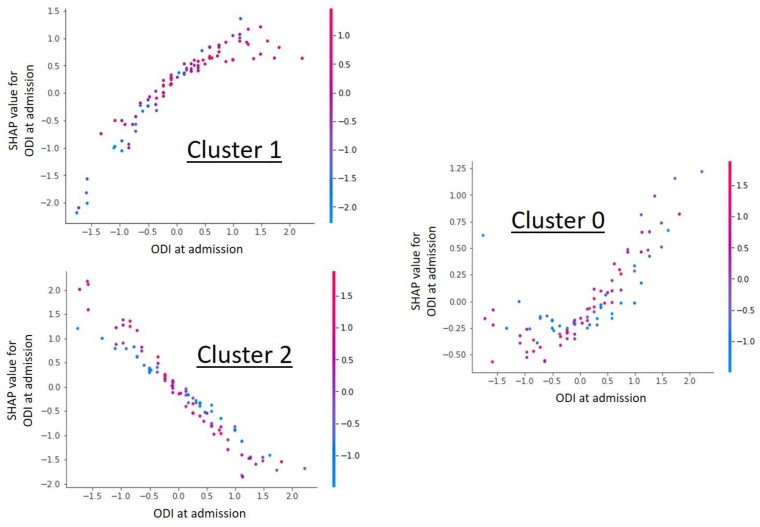
Shap value analysis of the parameter “ODI at admission” for each individual cluster. The graphs show a dependency scatter plot depicting the effect of the specific feature (ODI at admission) on the entire data set for a given cluster group. It can be clearly seen that the ODI value has cluster-specific different correlations and thus a high influence on cluster formation is likely.

In the framework of the cluster group analysis, the ODI at admission was shown to be the most important influencing factor on the cluster grouping (Figure 1) and at the same time showed a cluster specific correlation in the Shap value analysis (Figure 2). Further detailed analysis of the cluster groups revealed clinically relevant diagnosis subgroups within the main cluster groups that could be identified. To be able to use the tendencies of the ODI score on admission and at the end of therapy to differentiate between the diagnosis subgroups, only the 427 data sets with completely recorded ODI scores were used. The predicted ODI value at discharge was converted into a value that describes the change in ODI compared to the start of therapy.

Accordingly, the following data are available for an individual patient at the start of treatment:

- given ODI at admission;
- predicted change in ODI after therapy;
- predicted cluster group a patient belongs to.

If one analyzes the general ODI values in the predicted cluster group and compares them with the two individual ODI values of the patient (given at admission and predicted at discharge), it becomes possible to identify diagnostic groups based on the ODI characteristics of the patient.

## 3. Results

### 3.1. Prediction of Treatment Efficiency

The supervised AI model was performed in 5-fold cross-validation and achieved a mean absolute error of 9.06 in the prediction of the ODI value at discharge. A standard deviation of 0.17 was found (Table 2).

### 3.2. Unsupervised Cluster Analysis with Supervised Cluster Prediction

**Figure 3:**
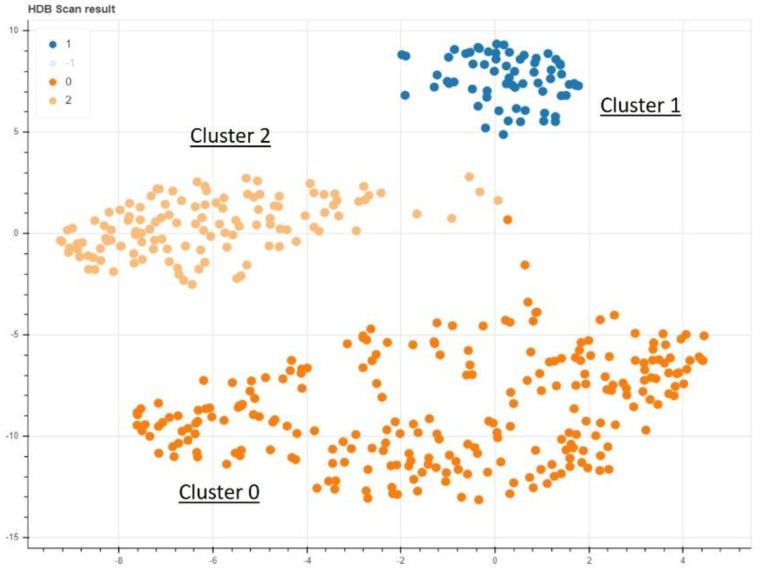
The nonsupervised data analysis shows a clear delineation of 3 different clusters.

**Figure 4:**
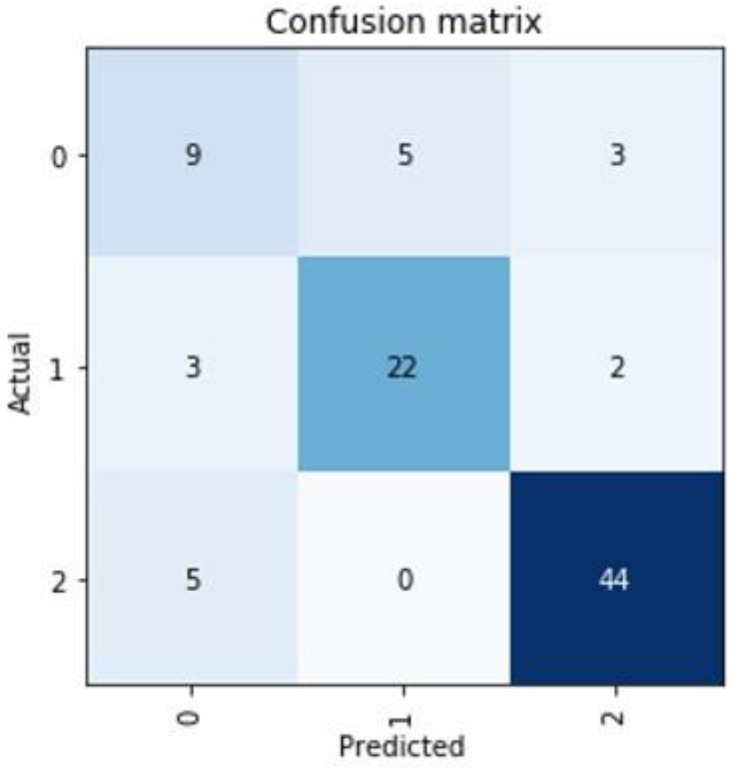
Representation of the worst prediction for the cluster group of a patient. Mainly in the cluster group “0” there seem to be difficulties in establishing a good prediction.

In the non-supervised AI model, a clear clustering with three separate areas could be found (Figure 3). Training a supervised model to learn the cluster labels assigned, we could achieve a mean of 89.54% prediction accuracy in a 5-fold cross validation approach with a standard deviation of 5.25% (Table 3). This cluster predicting model was found to be predominantly defined and determined by the ODI and VAS values of the patients at the time of admission (Figure 1). The cluster group “0” in particular proved to be an obstacle for a more optimal prediction, as a prediction failed here conspicuously often (Figure 4).

### 3.3. Combined AI Approach for Predicting Groups of Diagnoses

An analysis of the diagnosis subgroups in each cluster in Table 4 shows that these subgroups within a cluster can be distinguished from each other by certain combinations of ODI value at admission and change in ODI at discharge. In particular, certain pathologies stand out clearly in several cluster groups. In cluster “0”, a well recognizable constellation for herniated discs can be distinguished. This subgroup has unusually strong improvements in ODI value due to therapy. In addition, the subgroup of deformities in cluster “0” with low ODI values at the beginning of therapy and almost unchanged ODI values after conservative treatment can be well identified, too. In cluster group “1”, the diagnosis group of osteochondrosis stands out from the other subgroups with low initial ODI values and almost unchanged values after therapy. In cluster group “2”, tendencies for individual diagnostic groups are recognizable. Thus, osteoarthroses, osteochondroses and olistheses with insufficient changes in the ODI values following therapy distinguish themselves from the other subgroups.

## 4. Discussion

### 4.1. Prediction of Efficiancy of Conservative Treatment of Back Pain

Our supervised AI model for the prediction of ODI scores after conservative treatment can be considered useful to predict the success of conservative treatment of back pain. Unfortunately the absolute error rate of about 9 percentage points on the ODI scale does not lie within the minimum clinical difference (MCID) for the ODI which is also 9% (with 95% confidence interval) [17]. For the optimal quality of the assessment, a further reduction of the error rate below 9% would be required. However, whether or not an improvement of the patient’s complaints is to be expected through therapy, this can already be assessed indicatively with this AI model.

The reason for the insufficient prediction rate is not the number but the quality of the data [18]. We showed previously that a good prediction is possible even with small groups if the data quality is appropriate [19]. The present study is a preliminary study using prospectively collected registry data generated in daily clinical routine. There was no thorough data review and no explicit measures to ensure data quality. In line with current ideas on the establishment of artificial intelligence as discussed in the current literature, we see this as the main reason for the not entirely convincing predictive power of this AI model [2,3,4,18,20].

### 4.2. Prediction of Cause of Back Pain

In a separate analysis, we looked for a way to identify the causes of a patient’s back pain solely on the basis of the data set used here, without performing further diagnostics such as MRI or X-ray.

For this purpose, we first conducted a cluster analysis and then established a prediction of these clusters. Further analysis of the clusters led to subgroups that can be identified with the available predictions and baseline data of an individual patient.

To our knowledge, the interlinking of a cluster data analysis with a supervised AI for the prediction of cluster subgroups, represents a new approach for AI algorithms to address clinical questions. The diagnosis subgroup into which a patient can be assigned is identified in a kind of decision tree. This is built up from a combined supervised and non-supervised AI and provides distinguishing features based on the ODI on admission, the change in ODI after therapy, and the predicted cluster group. These features can be applied to the subgroups of clusters that are diagnosis-based, allowing the underlying diagnosis of a patient to be determined.

The correct cluster for a new patient is predicted with an accuracy of 89.54% in our model. The standard deviation here is 5.25%. These values show that the prediction is possible, but for clinical application, these values should be considered insufficient. From our point of view, an improvement of the data quality is a necessity for an improvement of the prediction, as stated above. In detail, when the dimensionally reduced cluster representation is combined with the detailed analyses of the individual predictions, it becomes apparent that the cluster group “0” probably consists of several individual clusters, but the available data sets cannot produce sufficient discriminatory power to differentiate them further. As a result, the predictions mainly show difficulty in correctly predicting the cluster group “0” (an example of the weakest prediction is shown in Figure 4).

Consequently, the diagnosis subgroup prediction based on the cluster prediction is also rudimentary in the present version. Clear differentiation is currently only possible for several diagnosis subgroups. Often only tendencies are recognizable in the current form, which make a definite classification into a diagnosis group difficult. It is well recognized that clinically related diagnostic groups also achieve comparable ODI values (Table 4). This confirms that the predictions basically work, however the required discriminatory power of the subgroups cannot, unfortunately, be achieved with the present data set.

### 4.3. Weaknesses of the Presented Concept

Since the data sets used here are the results of a single center and the therapies were carried out in all patients with the same components, we see the main problem to be the insufficient data quality. Secondly, the amount of data in this study could also be considered insufficient as the available data combine a large number of pathologies [21]. We have been able to show in a previous study that an assured data quality from the beginning leads to good predictions with regard to an AI establishment, even with small data sets.

Although it has been shown that imaging often does not necessarily correlate with the clinical impact of spinal changes [1], integration of existing image datasets should be considered in the further course provided it is not misunderstood to explain clinical symptoms. Particularly in determining a diagnosis, correlation with radiographs and/or MRI images is useful for quality reasons if only to verify the individual diagnoses in the dataset.

The data set of our methodological study used here has no relevant follow-up data. The predicted ODI value at the time of discharge after inpatient treatment is recorded approx. 5 days after the baseline values were recorded. To make a relevant statement about the success of the therapy, follow-up data would have to be collected several weeks to months after the treatment [17,22,23]. An accordingly adapted trial procedure has already been established for the further course of the study.

### 4.4. Artificial Intelligence in Treatment of Back Pain

The application of artificial intelligence algorithms in spine therapy is slowly gaining momentum. A few years ago, there were hardly any approaches to using the latest algorithms to optimize therapy for patients with back pain and spine related problems [20,24]. In the meantime, it has been shown several times that, using modern techniques, predictions can also be made for these patients with increasing accuracy [2,3,4,5].

Our presented concept shows that a combination of different AI techniques represents an added value and can be adapted towards clinical challenges. We believe our work can provide another component to further establish artificial intelligence in the treatment of back pain.

## 5. Conclusions

In this methodological study, we show how different artificial intelligence approaches can be applied in a patient collective, both individually and in combination, to gain insights into the cause of back pain in individual cases.

## Figures and Tables

**Table 1:** Variables used in AI programming.

Categorical Variables	Continuous Variables	Target Variable
Gender	Age	ODI at dismission
Pain duration > 3 month	Year of birth	
Smoking	BMI	
Previous spine operation	Back pain (VAS)	
	Leg pain (VAS)	
	ODI at admission	
	Patient standing height	

**Table 2:** Mean absolute error rate and mean and standard deviation of the performed 5-fold cross-validation for the prediction of the ODI Score at dismission.

Fold	1	2	3	4	5	Mean	SD
Mean absolute error	8.91	8.94	8.99	9.29	9.20	9.06	0.17

**Table 3:** Mean correct prediction rate of the cluster group and mean and standard deviation of the performed 5-fold cross-validation.

Fold	1	2	3	4	5	Mean	SD
correct prediction	89.4%	80.6%	92.3%	91.5%	93.9%	89.54%	5.25%

**Table 4:** Mean values of ODI at admission (ODI initial) and change in ODI value at the time of discharge from hospital (with standard deviation in grey beneath) for the diagnosis subgroups in all three clusters. In the overall comparison, higher values are highlighted in yellow and lower values in blue.

	Cluster 0			Cluster 1			Cluster 2		
	n	ODI Initial	Delta ODI	n	ODI Initial	Delta ODI	n	ODI Initial	Delta ODI
osteoarthritis	27	46.39	6.24	2	46.11	16.78	16	29.11	5.82
		12.39	4.10		3.89	1.22		11.75	4.16
deformity	14	30.90	3.34				1	26.00	19.33
		5.96	3.04					0.00	0.00
osteochondrosis	74	45.67	7.53	6	22.93	3.44	22	27.94	5.93
		15.84	8.07		11.45	4.99		11.62	6.63
olisthesis	10	47.10	6.13				4	28.00	6.78
		15.34	4.20					8.71	2.03
other degenerative	20	50.79	8.51	8	46.50	11.57	12	30.87	11.36
		13.70	6.25		5.70	6.61		8.83	9.33
spinal stenosis	55	51.09	9.99	5	49.96	3.33	13	27.77	12.05
		16.41	9.61		6.20	3.96		10.21	9.46
disc herniation	24	49.07	21.77	32	47.34	3.19	45	35.34	15.67
		19.60	16.71		14.34	4.72		11.53	13.04
other radiculopathy	26	52.00	3.02	2	55.00	5.00	9	32.20	8.89
		14.72	2.99		3.00	5.00		14.49	12.83

## Data Availability

The algorithms and original data sets used in this study can be reviewed upon reasoned request via the corresponding author.
